# Is Lumbosacral Transitional Vertebra Associated with Degenerative Lumbar Spinal Stenosis?

**DOI:** 10.1155/2019/3871819

**Published:** 2019-06-10

**Authors:** Janan Abbas, Natan Peled, Israel Hershkovitz, Kamal Hamoud

**Affiliations:** ^1^Department of Anatomy and Anthropology, Sackler Faculty of Medicine, Tel Aviv University, Tel Aviv 6997801, Israel; ^2^Department of Physical Therapy, Zefat Academic College, Zefat 13206, Israel; ^3^Department of Radiology, Carmel Medical Center, Haifa 3436212, Israel; ^4^Faculty of Medicine in the Galilee, Bar-Ilan University, Zefat 1311502, Israel; ^5^Department of Orthopaedic Surgery, The Baruch Padeh Poriya Medical Center, Tiberias 1520800, Israel

## Abstract

The aim of this study was to shed light on the association between lumbosacral transitional vertebra (LSTV) and degenerative lumbar spinal stenosis (DLSS). A cross-sectional retrospective study was performed on 165 individuals that were diagnosed with clinical picture of DLSS (age range: 40-88 years; sex ratio: 80M/85F) and 180 individuals without DLSS related symptoms (age range: 40-99 years; sex ratio: 90M/90F). All participants had undergone high-resolution CT scan for the lumbar region in the same position. We also used the volume rendering method to obtain three-dimensional CT images of the lumbosacral area. Both males and females in the stenosis group manifest greater prevalence of LSTV than their counterparts in the control group (P<0.001). Furthermore, the presence of LSTV increases the likelihood of degenerative spinal stenosis (odds ratio= 3.741, P<0.001). In the control group, LSTV was more common in males, and sacral slope angle of males was significantly greater in LSTV group compared to non-LSTV. This study indicates that LSTV was significantly associated with symptomatic DLSS.

## 1. Introduction

Lumbar spinal stenosis is one of the most commonly diagnosed and treated conditions among the elderly population [1, 2]. Its clinical prevalence is about 47% in adults with lower extremities symptoms and 13% in those who seek help from a specialist for low back pain (LBP) [3–5]. Degenerative lumbar spinal stenosis (DLSS) is considered the most common acquired type [6] and is associated with degenerative changes of the three-joint complex, ligamentum flavum thickening, and osteophytes formation [7–9].

Lumbosacral transitional vertebrae (LSTV) are common congenital spinal anomalies, referring to a total or partial unilateral or bilateral fusion of the transverse process of the lowest lumbar vertebra to the sacrum [10]. Generally, the term LSTV is used to avoid having to decide whether the vertebra is sacralized L5 or a lumbarized S1 because it is not possible to view the entire spine [10]. Their reported prevalence range between 4% and 36% [11–14] with a remarkable preference in men [15, 16]. It has been reported that LSTV are generally easier to detect on CT images than on magnetic resonance imaging (MRI) [17, 18].

Although several studies have correlated the presence of LSTV with LBP and nerve-root symptoms [10, 19–21], other investigators have disputed this [13, 22–25]. In addition, some studies have noted that LSTV might increase the risk for developing lumbar spine degeneration at the level above the transitional vertebra [23, 26] in LBP individuals; however, data regarding LSTV and DLSS are ambiguous. Additionally, the association between LSTV and lumbar curvatures is uncommon [27, 28].

The aims of this study were (1) to identify the prevalence of LSTV in symptomatic DLSS, (2) to reveal whether the presence of LSTV affects disc height at the level above the LSTV, and (3) to examine the association between LSTV and lumbar lordotic curvatures.

## 2. Materials and Methods

### 2.1. Study Design

This is a cross-sectional retrospective study with two groups of individuals [29]. The first group (control) included 180 individuals without spinal stenosis related symptoms (age range: 40-99 years; sex ratio: 90M/90F). This group was randomly collected (2008 to 2010) from a pool of subjects referred to the Department of Radiology, Carmel Medical Center, Haifa, Israel, for abdominal CT scans due to abdominal problems. The second group included 165 patients with symptomatic DLSS (age range: 40-88 years; sex ratio: 80M/85F), who were enrolled from 2006 to 2010 and had intermittent claudication accompanied by other symptoms related to spinal stenosis (LBP and radicular pain) [30, 31]. The CT scans of these patients showed a reduced cross-sectional area (CSA) of the dural sac (<100 mm^2^) [32–34] of at least one lumbar level. The diagnostic criteria for DLSS were based on the combination of symptoms and signs together with the imaging findings [35]. Individuals under 40 years of age as well as those with congenital stenosis (AP diameter of the bony canal < 12 mm) [36, 37], fractures, spondylolysis, tumors, Paget's disease, steroid treatment, severe lumbar scoliosis (>20 degrees), and iatrogenic conditions (after laminectomy, after fusion) were excluded from the study.

A high-resolution CT image (Brilliance 64, Philips Medical Systems, Cleveland, OH; slice thickness 0.9–3mm, voltage 120 kV, current 150–570 mA) was utilized which enabled scan processing in all planes and allowed a 3D reconstruction of the lower lumbar region. All CT images for both groups were taken in the supine position with extended knees.

This study was approved by the ethical committee of the Carmel Medical Center (0083-07-CMC).

### 2.2. Identification of LSTV

**Figure 1:**
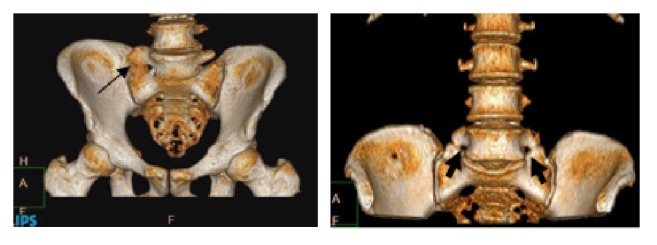
Lumbosacral transitional vertebra as evident in 3-dimensional images: unilateral (left) and bilateral (right) anomalies.

The presence of LSTV was based on Castellvi classification system [10] using the volume rendering method to obtain three-dimensional CT images of the lumbosacral area (Figure 1). The definition of LSTV was performed by the first author (JA) under the supervision of a diagnostic radiologist. The participants with positive LSTV were then recorded into unilateral or bilateral anomalies regardless of the severity of LSTV.

In this study, the disc level above the LSTV was related to the segment between the last lumbar vertebra and the sacrum, irrespective of whether the LSTV was a sacralized L5 or a lumbarized S1 (following the study of Otani et al.) [23].

### 2.3. Sacral Slope Angle (SSA)/Lumbosacral Angle

 SSA was measured in the mid-sagittal plane, using a modification of Ferguson's method [38] (adapted to CT images when the individual is in a supine position) and defined as the angle formed by the line of the upper end plate of the sacrum and the horizon.

### 2.4. Lumbar Lordosis Angle (LLA)

 LLA was evaluated in the sagittal plane between the lines of upper endplate of L1 and S1 following Cobb's method [39] (adapted to the sagittal plane).

### 2.5. Intervertebral Disc Height (IDH)

 IDH was measured in the mid-sagittal plane at three points: anterior, middle, and posterior. Mean IDH was then calculated for the three different locations.

### 2.6. Statistical Analysis

The sample size of this study was based on the power analysis (*α*= 0.05, *β*=0.8) and all the statistical analyses were done via SPSS version 20. Chi-Square test was performed to compare the prevalence of LSTV between the study groups (control and stenosis) for each gender separately. A logistic regression analysis was also used to determine the association between DLSS and LSTV (dependent variable: DLSS; independent variables: LSTV, age, gender, BMI) using “Forward LR” method. To identify the relationships between LSTV with lumbar curvatures and/or disc height we used t-test for each gender separately for the control group (adjusted for age and BMI).

Kappa and intraclass correlation (ICC) coefficients were calculated to determine the intratester and intertester reliability of LSTV and the metric parameters, respectively (repeated measurements of 20 individuals). Intratester reliability was assessed by one of the authors (JA) who identified the LSTV presence twice within intervals of 3-5 days. Intertester reliability involved two testers (JA and KH), who took the measurements within an hour of each other. Both testers were blinded to the results of the measurements. Significant difference was set at P < 0.05.

## 3. Results

Kappa coefficient tests for both intra- and intertester reliability were very high, 0.990 and 0.980, respectively. In addition, the intraclass correlation coefficient (ICC) test for intratester and intertester reliability of the parametric variables (e.g., disc heights and lumbar curvatures) ranged from ICC= 0.960 to 0.984 and from ICC= 0.943 to 0.980, respectively.

Data for age and body mass index (BMI) of both study groups (control vs. stenosis) is presented in Table 1.

### 3.1. Prevalence of LSTV in the Study Groups

We found that 95 individuals who manifest spinal stenosis (57.6%) have LSTV (unilateral and bilateral together) compared to 47 (26.1%) in the control group (P<0.001).

**Figure 2:**
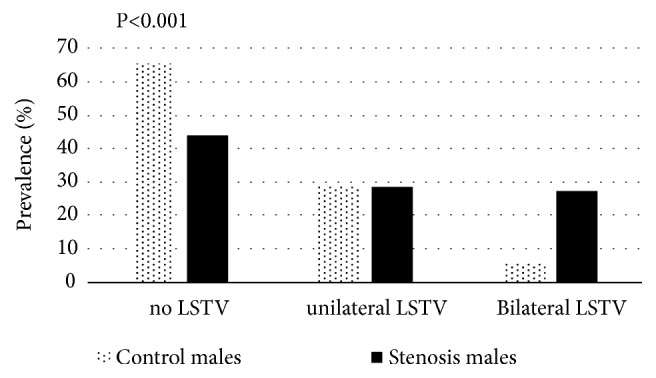
Prevalence (%) of lumbosacral transitional vertebra (LSTV) in the male groups (control vs. stenosis).

**Figure 3:**
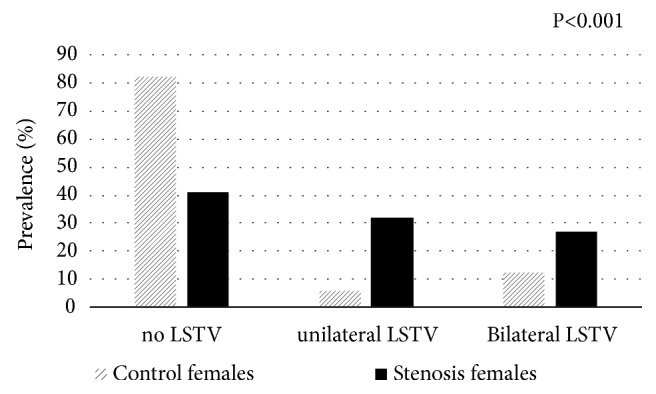
Prevalence (%) of lumbosacral transitional vertebra (LSTV) type in the female groups (control vs. stenosis).

The prevalence of LSTV for each anomaly (unilateral and bilateral separately) in both stenosis males and females was significantly higher compared to their counterparts in the control group (P<0.001) (Figures 2 and 3). In the stenosis group, there were no significant differences in the degree of stenosis (according to cross-sectional areas of dural sac from L1-2 to L5-S1) between the unilateral and bilateral LSTV.

Furthermore, the presence of LSTV (unilateral and bilateral combined) was found to increase the likelihood of DLSS development (odds ratio= 3.741, confidence intervals=2.342-5.974, P<0.001) (Table 2). However, logistic regression analysis for bilateral and unilateral LSTV separately showed different OR (odds ratio): OR = 5.451, CI (confidence intervals) = 2.820-10.535, P<0.001; OR = 2.889, CI = 1.666-5.008, P<0.001, respectively.

### 3.2. Association between LSTV and Gender, Lumbar Curvatures, and Disc Height

Among those who manifest LSTV in the control group (n=47) we found 31 males and 16 females (66% vs. 34%, P=0.017).

As considerable differences in mean age between the LSTV and non-LSTV individuals in the control females group have been reported (69.6 ± 11.3 vs. 60.4 ± 12.7; P=0.009, respectively), we reduced the sample size of the females non-LSTV (from 74 to 62) to avoid age bias (Table 3).

In males, the mean disc height of the supradjacent level to LSTV was significantly smaller compared to the same levels in individuals without LSTV. Additionally, lumbar curvatures were greater in individuals with LSTV compared to non-LSTV group, yet significant difference was noted only for sacral slope (Table 3). In females, however, neither disc height nor spine curvatures were associated with LSTV.

## 4. Discussion

As this study, to our knowledge, is the first to establish the prevalence of LSTV in symptomatic DLSS, it could not be debated with others.

Symptomatic lumbar spinal stenosis requires appropriate specific history and physical examination findings combined with radiographic findings [30]. Neurogenic claudication and radicular pain constitute the best described clinical picture while neurogenic claudication is the most common one. This symptom is a variable pain or discomfort with walking or prolonged standing that radiates beyond the spinal area into one or both buttocks, thighs, lower legs, or feet [35]. It also exhibits typical provocative features, such as improvement with sitting or lumbar flexion, and worsening with lumbar extension [30, 35]. In contrast, radicular pain may often not exhibit the provocative features seen in neurogenic claudication. Furthermore, low back pain is often present and its actual part in this syndrome is controversial [35].

Our result indicates that the prevalence of LSTV in the DLSS group (57%) is about 2 times greater than the control group (26%) and is much higher than the previous reported studies (range: 4-36%) that based their investigation on LBP individuals as well as healthy and general population [14–16, 22, 24, 40–42]. We believe that the wide range of this reported incidence could likely be due to differences in individual diagnostic and classification criteria, observer error, imaging techniques, and other confounding factors of the studied population [43]. It is noteworthy that the prevalence of LSTV for the control group (26%) falls within the range reported by other studies (16-30%) that were conducted on general population [14, 16, 22].

This study revealed that stenosis males and females manifest greater prevalence of LSTV compared to the control. Additionally, the presence of LSTV increases 3.741 times the risk of developing DLSS. Although Elster (1989) stated that spinal stenosis and nerve-root canal stenosis were much more common at the level immediately above a transitional vertebra than at any other level [21], the correlation between spinal stenosis and LSTV was refuted [21, 44].

It is well-known that one of the main roles of the lumbosacral region is distributing the load from the entire lumbar spine to the hip joints and then to the lower limbs [45]. The transmitted load that passes through the lumbosacral joints includes the three-joint complex such as the intervertebral disc, anteriorly, and 2- facet joints, posteriorly. Mahato (2012) reported that LSTV has the potential to alter the biomechanics of lumbar spine as many of these transitions manifest deformities of the zygapophyseal surfaces and hence cause listhesis at L5-S1 [46]. Furthermore, unilateral LSTV results in asymmetrical biomechanical alterations that can cause facet pain [47] and influence disc degeneration [27]. In anatomical cadavers study, Aihara et al. (2002) found that iliolumbar ligaments above the transitional vertebrae were thinner and weaker than those without LSTV [48]. They suggested that this result was a response to vertebral instability that could subsequently lead to segmental degeneration. It has been also proposed that in the presence of sacralization of L5, when its transvers process is fused or anomalously articulated with the sacrum, motion of L5-S1 segment is restricted [49], which could lead to excessive movements of the upper segment, similar to the adjacent segment pathology after a spinal fusion [50]. Mahato (2013) found that the overall number of trabeculae was reduced in sacralized L5 vertebra, which may emphasize the altered trajectory stress on the lumbosacral junction [51].

We believe that the association between LSTV and DLSS is related to the fact that LSTV increases the mobility and alters the mechanical stress above the transient vertebra that may lead over time to degenerative changes of the three-joint complex [52] causing stenosis. Furthermore, hypermobility and abnormal torque movements at the level above the transitional vertebra have been reported [22, 53].

We also found that disc height loss (male groups) above the transition level was significantly associated with LSTV. We assume that the insignificant result obtained for females is due to low number of subjects with LSTV anomaly (n=16). Disc protrusion and/or extrusion occurs more often at the level supradjacent to the LSTV than the same level in patients without LSTV [10, 21, 26]. This is also true for disc degeneration [26, 54]. Otani et al. reported that 83% of patients with disc herniation in the presence of LSTV experience symptoms arising from the last caudal mobile segment, whereas 59% of patients with disc herniation without transitional vertebra had arising symptoms from the 2nd last mobile segment [23]. Furthermore, a recent study [49] has found that among adolescent patients with sacralization, the L4-5 disc herniation was significantly more common than L5-S1 (81.3% vs. 18.7%, P=0.019).

Our results showed that LSTV in the control group has great preference in males than females (66% vs. 34%, P=0.017), which is in agreement with previous reports [10, 15, 16, 55]. In contrast, other studies stated no statistical correlation between LSTV and gender [56, 57]. Genetic factors are considered to be responsible for the segmentation development of lumbosacral spine [11]. Uçar and colleagues suggested in their wide and well-represented population study (n=3607) that the great LSTV prevalence among males could be a part of body size gender dimorphism in human beings [16]. We assume that the relation between gender and LSTV could partially explain why male lumbar discs (besides other factors, e.g., occupation) were significantly more degenerated than female discs across most ages [58].

We found that sacral slope has a tendency to be greater among LSTV individuals compared to non-LSTV; however, significant difference was noted only for males (44.4± 7 vs. 40.8 ± 6, P=0.048). Although the influence of LSTV on sacral anatomy and lumbar curvature has been less studied in the past, our result is in accordance with some previous studies. Chalian et al. found that both sacral slope and lumbar lordosis were significantly increased in LSTV compared to controls and this finding supports their previous experiences [27]. One study has also reported that L5-S1 accessory articulations were characterized with increased lordotic curves [59]. In contrast, a recent study that was conducted in individuals with LBP reported that sacral tilt was significantly smaller in LSTV than those without LSTV [28]. We believe that the differences between the studied populations of the current study and the latter one (general population vs. LBP) may explain the opposite result since LBP could affect lumbar spine curvatures [60–62].

The increased sacral slope in individuals with LSTV could lie within the potential effect of vertebral transition upon the normal biomechanics of lumbar spine. This means that modification of sacral slope in individuals with LSTV could be related to the alteration of trajectories applied upon lumbar spine. As mentioned above, disc height supradjacent to the LSTV tends to be reduced [26, 54]. Furthermore, attenuated L5 vertebral heights in individuals with L5-S1 fusion have been reported [59]. Therefore, reduction of the spine length is expected, resulting in increased lumbar curvature due to diminishing of Delmas index [63].

### 4.1. Limitation of the Study

This is a retrospective research and the outcomes should be supported by well-established prospective studies. Additionally, the sample size of the control group was relatively small and a large-scale population with LSTV is needed to shed light on this phenomenon and to reveal its association with lumbar spine alterations.

## 5. Conclusions

The current study shows that the prevalence of LSTV is significantly greater in the stenosis group compared to control. The presence of LSTV may increase the likelihood of developing DLSS; however, no causal relationship is reported. The LSTV is gender-dependent and may exaggerate sacral curvature.

## Figures and Tables

**Table 1:** Age and body mass index values of the study groups (control vs. stenosis) for each gender separately.

Variables	*Males*			*Females*		
	Control (mean±SD)	Stenosis (mean±SD)	P value	Control (mean±SD)	Stenosis (mean±SD)	P value
Age (years)	62.9±12.38	66.2 ±10.82	0.066	62 ±12.97	62.5 ± 8.63	0.795

BMI (kg/m^2^)	27.4 ±4.21	28.9 ± 4.55	0.021	27.61±5.13	31.48±5.83	<0.001

SD: standard deviation.

**Table 2:** A logistic regression analysis demonstrating the variables that significantly associate with degenerative lumbar stenosis.

Variable	OR	(CI) 95%	P value
BMI	1.112	1.061-1.116	< 0.001
LSTV	3.741	2.342- 5.974	<0.001

LSTV: lumbosacral transitional vertebra, OR: odds ratios, CI: confidence intervals, BMI: body mass index.

**Table 3:** Mean age, sacral slope (SS), lumbar lordosis (LL), and intervertebral disc height (IDH) in the LSTV and non-LSTV for the control group.

	Non-LSTV Mean ± SD	LSTV Mean ± SD	P value
*Males*	(n=59)	(n=31)	
Age	61.5 ± 12	65.6 ± 11	0.128
BMI	27.3 ± 4.5	27.6 ± 3.5	0.704
SS	40.8 ± 6	44.4 ± 7	*0.048*
LL	48.2 ± 8	52.4 ± 11	0.089
IDH	9.7 ± 2	7.8 ± 2	*<0.001*

*Female*	(n=62)	(n=16)	
Age	63.6 ± 11	69.6 ± 11	0.071
BMI	27.6 ± 5	27.5 ± 5.6	0.915
SS	41.1 ± 7	45.6 ± 8	0.076
LL	51.4 ± 12	55.1 ± 11	0.253
IDH	8.6 ± 2	8.4 ± 2	0.765

SD: standard deviation, n: sample size, LSTV: lumbosacral transitional vertebra.

## Data Availability

The data used to support the findings of this study are available from the corresponding author upon request.
